# Assessing erythroferrone and iron homeostasis in preeclamptic and normotensive pregnancies: A retrospective study

**DOI:** 10.1016/j.placenta.2023.01.008

**Published:** 2023-01-20

**Authors:** Zahra Masoumi, Lucas R. Hansson, Eva Hansson, Evelina Ahlm, Eva Mezey, Lena Erlandsson, Stefan R. Hansson

**Affiliations:** a Division of Obstetrics and Gynecology, Department of Clinical Sciences Lund, Lund University, Lund, Sweden; b Adult Stem Cell Section, National Institute of Dental and Craniofacial Research, National Institutes of Health, Bethesda, USA; c Skåne University Hospital, Obstetrics and Gynecology, Sweden

**Keywords:** Erythroferrone, iron homeostasis, preeclampsia, placenta

## Abstract

**Introduction:**

Preeclampsia (PE) is a pregnancy-related disorder associated with maternal hypertension and placental dysfunction. A significant micronutrient during pregnancy is iron, which is important in cellular functions. While iron absorption increases in pregnancy, little is known about the exact mechanisms regulating maternal iron levels and transfer through the placenta in normal and complicated pregnancies.

**Methods:**

In this retrospective study, we investigated the regulation of maternal and placental iron availability and storage, in normotensive and pregnancies complicated by early- or late-onset PE. Methods used were analysis of clinical records, ELISA analysis on plasma samples, immunofluorescent and Prussian Blue analysis on placenta biopsies.

**Results:**

Focusing on erythroferrone (ERFE) as a new marker and hormonal regulator of iron, our results demonstrated altered maternal ERFE levels in PE. We are the first to report the expression of ERFE in trophoblasts and indicate its lower levels in early-onset PE placentas. These changes were associated with lower placental transferrin receptor 1 (TfR1) in syncytiotrophoblasts in both early- and late-onset PE. In addition, maternal plasma ERFE levels were elevated in both early- and late-onset PE and hepcidin levels reduced in early-onset PE. Unaltered maternal plasma IL-6 levels suggest mechanism other than inflammation being involved in altered iron regulation in PE pregnancy.

**Discussion:**

Our data supports a deregulation in maternal iron bioavailability in early- and late-onset PE vs normotensive pregnancies. The exact role of placental ERFE in regulating maternal-placental-fetal iron transport axis requires further investigation.

## 1. Introduction

Preeclampsia (PE) is a pregnancy disorder diagnosed based on maternal hypertension in combination with evidence of organ damage and/or uteroplacental dysfunction presenting after 20 weeks of gestation [1]. The disease is further defined as early-onset PE (delivery before 34 gestational weeks (GW)), that involves a defect placentation often causing fetal growth restriction and more severe maternal manifestations compared to late-onset PE [2]. The exact underlying mechanisms of PE remain elusive, but it is generally accepted that there are two stages of PE pathogenesis, where impaired invasion of trophoblasts into the decidua and inadequate remodeling of the spiral arteries is the first step. This leads to defective utero-placental circulation, hypoxia, oxidative stress, syncytiotrophoblast stress and placental dysfunction in PE [3,4]. These events are more considered to be associated with the development of early-onset PE and affects 20% of all pregnancies. Development of late-onset PE, that accounts for majority of cases, is more often associated with preexisting maternal conditions and risk factors such as obesity, diabetes, kidney disease and autoimmune diseases, several characterized by increased inflammation and endothelial dysfunction. The clinical manifestations seen in the second stage become apparent after 20 weeks of gestation and are shared by early- and late-onset PE [1]. Defective utero-placental circulation results in altered maternal physiological adaptation to pregnancy and lower blood volume expansion [5], which in turn leads to hemoconcentration and venous congestion as seen in PE pregnancies [6].

Iron is an essential element and central to various cellular processes including oxygen transport, oxidation-reduction reactions in the electron transport chain and DNA synthesis [7]. Due to its high redox activity, accurate regulation of iron is crucial to avoid Fenton reaction that leads to massive production of reactive oxygen species. These regulatory mechanisms gain further importance during pregnancy when an additional ~900 mg of iron is required to allow for placental perfusion as well as fetal erythropoiesis and maternal blood volume expansion [8]. A portion of the additional iron is found as granules in the placental syncytium, that gradually decrease over gestation and are altered in abnormal pregnancies, such as molar pregnancies and fetal growth restriction [8,9]. Iron is stored bound to ferritin, the major iron storage molecule found in most tissues, making it a valuable marker for iron storage. Increased serum iron and ferritin levels have been studied in PE [8–10], and hyperferritinemia seen in PE has been associated with adverse fetal outcomes [11]. However, the findings in the literature are inconsistent [8–10] and the exact mechanisms regulating iron homeostasis in pregnancy [12], particularly those complicated by PE, remain to be studied.

Hepcidin [13] and erythroferrone (ERFE) [14] are both involved in the regulation of iron homeostasis. Hepcidin is mainly expressed by liver hepatocytes and decreases iron availability by limiting absorption of dietary iron in the gastrointestinal tract and inhibiting mobilization of iron stores [15]. Erythroferrone, the other regulatory hormone identified only in 2014 [14], is expressed primarily by erythroblasts and increases plasma iron availability by suppressing hepcidin to facilitate hemoglobin and erythropoiesis [16]. Recent *in vitro* and *in vivo* studies suggest that fetal and placental hepcidin levels are very low, and that instead maternal hepcidin levels regulate both fetal and maternal iron availability and storage during pregnancy [17–19]. Suppression of maternal hepcidin in a healthy pregnancy leads to higher iron absorption from the diet and release of iron from maternal iron stores [18,19]. This increases bioavailable transferrin (Tf)-bound iron for placental uptake via transferrin receptor 1 (TfR1) expressed on the apical surface of the placental syncytium [20,21]. The iron is then transported to placental stroma and fetal circulation via ferroportin (FPN), located in the basolateral membrane of the syncytiotrophoblasts [20,21]. Interestingly, while hepcidin has been the focus of extensive research [18, 19], maternal levels of ERFE in pregnancy or its placental expression have not been studied. The aim of this retrospective study was to investigate ERFE as an indicator of changes in maternal and placental iron homeostasis and storage in normotensive and PE pregnancies.

## 2. Material and method

### 2.1. Patient demographics and ethical approval

This is a retrospective study, using available patient data and samples (plasma and placenta biopsies) from a preexisting biobank created for research on PE at the Department of Obstetrics and Gynecology, Lund University, Sweden. Depending on availability of clinical information, plasma and/or placenta biopsies, the patients/samples were included to be used in different analyses. The study was approved by the Ethics Committee Review Board for studies on human subjects at Lund University and Skåne University Hospital, Lund, Sweden (Dnr 2014/191). The samples were collected from normotensive and PE pregnancies following Cesarean section or vaginal deliveries at Skåne University Hospital. Written informed consent was obtained from all participants. Preeclampsia was defined as blood pressure ≥140/90 mmHg, proteinuria ≥300 mg/L or other organ dysfunction, in accordance with guidelines used at the time of collection [22]. Early-onset PE was defined as delivery before or at 34 GW and late-onset PE as after 34 GW. Time of delivery was used instead of time of onset, since the exact time point for onset is less accurately documented at the antenatal visits and may occur before women present at the hospital. The placenta and/or maternal blood collected in an EDTA tube were stored at 4 °C and processed within 4 h after delivery. Additional clinical data indicating maternal iron status, such as maternal ferritin or hemoglobin levels, documented iron supplementation during pregnancy, as well as available Doppler analysis of the uterine and umbilical arteries were obtained from patient records. Fetal weight was evaluated and Small for Gestational Age (SGA) defined as weight deviation lower than 2 standard deviations, according to Niklasson et al. [23]. Summaries of the clinical demographics of the patients included in each set of analysis are listed in Tables 1–3 and Supplementary Tables S1–S3.

### 2.2. Maternal plasma collection and lactate dehydrogenase (LDH) ELISA

The maternal blood sample was centrifuged at 2000×*g* for 20 min. The uppermost layer containing maternal plasma was collected and stored at −80 °C until further analysis. To evaluate high LDH activity associated with hemolysis, a colorimetric lactate dehydrogenase (LDH) assay was performed using LDH assay kit (ab102526, Abcam) for 30 min according to the manufacturer’s protocol.

### 2.3. Ferritin immunoassay

Analysis of maternal plasma ferritin levels at time of delivery was performed at the Clinical Biochemistry Laboratory at Lund University Hospital, Sweden, using an Atellica IM Ferritin assay on an Atellica IM analyzer (Siemens Healthcare Diagnostics Inc.). The analysis is a sandwich method using chemiluminescence and two anti-ferritin antibodies, one polyclonal goat anti-ferritin antibody conjugated to acridinium ester, and one monoclonal mouse anti-ferritin conjugated to paramagnetic particles. The level of chemiluminescence is proportional to the ferritin concentration.

### 2.4. IL-6 ELISA

Analysis of maternal plasma IL-6 levels at time of delivery was performed using a human IL-6-specific ELISA (Invitrogen) according to manufacturer’s protocol, and all samples were run as duplicates.

### 2.5. Hepcidin ELISA

Analysis of maternal plasma hepcidin levels at time of delivery was performed using the Human Hepcidin Quantikine ELISA kit (R&D Systems) according to manufacturer’s protocol, and all samples were run as duplicates.

### 2.6. Erythroferrone ELISA

Maternal plasma ERFE levels were measured using the Intrinsic Erythroferrone IE^™^ ELISA kit (ERF-001, Intrinsic Life Sciences) at room temperature (RT). In summary, ERFE standards were prepared, and samples were diluted in sample diluent at 1:2 ratio. 100 μl of standard or sample was added to each well of the microwell assay plate coated with anti-erythroferrone antibody, in duplicate, and the plate was incubated on an orbital shaker at 350 x rpm for 1 h. Following three rinses with wash solution, 100 μl of HRP Conjugate solution was added to each well and the plate was incubated on the orbital shaker for 30 min. After three rinses with wash solution, 100 μl of TMB substrate was added to each well and the plate was incubated in the dark for 20 min. The reaction was stopped by addition of Stop Solution and absorbance was measured at 450 nm using a GloMax^®^ Discover plate reader (Promega).

### 2.7. Transferrin receptor 1 ELISA

Maternal plasma TfR1 levels were analyzed using Human TfR/Transferrin R/CD71 ELISA Kit (EH448RB, Invitrogen^™^) at RT. In brief, standards were prepared, and samples were diluted in Assay Diluent D in 1:200 ratio. 100 μl of standard or sample was added to each well of the microwell assay plate coated with anti-TfR1 antibody, in duplicate, and the plate was incubated for 2.5 h. After four rinses with Wash Buffer, the samples were incubated with biotin-conjugate for 1 h with gentle shaking. Following four rinses with Wash Buffer, 100 μl of Streptavidin-HRP solution was added to each well and the samples were incubated for 45 min with gentle shaking. The wells were rinsed four times with Wash Buffer before adding 100 μl of TMB substrate to each well and incubating the plate in the dark for 30 min with gentle shaking. 50 μl of Stop Solution was added to each well to stop the reaction prior to measuring absorbance at 450 nm using a GloMax^®^ Discover plate reader (Promega).

### 2.8. Immunofluorescent analysis of placental transferrin receptor 1, erythroferrone and ferroportin

Analysis of placental expression of TfR1, ERFE and FPN was performed on a total of 10 normotensive, 10 late-onset PE, and 6 early-onset PE placenta samples (Table 3). All procedures were performed at RT unless stated otherwise. The placenta tissue biopsies were cut from an area within a 7 cm radius of the umbilical cord, rinsed in PBS and fixed in 4% buffered formaldehyde solution (Histolab^®^) for 24 h before paraffin embedding and sectioning. The sections (4 μm) were incubated at 60 °C for 1 h, deparaffinized in xylene and rehydrated in decreasing concentrations of ethanol. Antigen retrieval was performed in slow boiling 10 mM citrate buffer (pH = 6.0) for 20 min. After cooling to RT, the sections were permeabilized in 0.05% TritonX-100 in PBS (PBST) for 3 min, before blocking non-specific protein binding sites (Dako, X0909, Agilent) for 20 min. After a rinse in PBST, the samples were incubated overnight at 4 °C with appropriate primary antibodies against TfR1 (1:2000, ab84036, Abcam), ERFE (1:50, ab222468, Abcam) or FPN (1:150, NBP1–21502, Novus Biologicals) diluted in PBST containing 1.5% BSA and 1.5% FBS. Following 2 h incubation at RT and three 3-min rinses in PBST, the samples were incubated with a goat-anti-rabbit Alexa Fluor 488-conjugated secondary antibody (1:600, A11070, Invitrogen) in the dark for 1 h. Negative control test was included in each analysis by incubating a section without the primary antibody and following it by the secondary antibody incubation. The sections were further rinsed 3 times, each for 3 min, in PBST before mounting with ProLong^™^ Diamond Antifade Mountant with DAPI (P36962, Invitrogen^™^) and incubated in the dark overnight prior to microscopy. All negative control images are shown in supplementary material; TfR1 (Fig. S1A), FPN (Fig. S1B) ERFE (Figs. S1C and D).

### 2.9. Prussian blue staining

To analyze placental ferric iron storage, Prussian blue staining was performed. Paraffin-embedded placenta sections cut at 4 μm thickness were deparaffinized as mentioned above. The sections were incubated with freshly prepared solution containing equal parts of 20% hydrochloric acid and 10% potassium hexacyanoferrate (II) trihydrate (P3289, Sigma-Aldrich) (in dH_2_O) for 20 min. After three rinses in dH_2_O, nuclear counterstaining was performed by incubating the samples in nuclear fast red (AS-RRSP456-E, Histolab^®^) for 5 min. Following two rinses in dH_2_O, the sections were dehydrated and mounted with PERTEX^®^ (Histolab^®^) prior to microscopy.

### 2.10. Semi-quantification and grading of the microscopy analysis

Multiple images were obtained for each sample from the placental immunofluorescence analysis of TfR1, ERFE, and FPN, and were saved in.tiff format. The images were semi-quantified using Fiji (Open-source platform, ImageJ) and a method previously published [24]. In summary, a duplicate of an image from the green fluorescent-channel was used to set threshold and create a mask that would specifically cover the syncytium or the area containing placental bed giant cells, a sub-population of extravillous trophoblast cells [25]. These areas were selected and added to the ROI manager in Fiji, along with an area of 30 × 30 μm of the background with no tissue. The signal and background from the green channel were then measured. After deducting the average background from the average signal for each specific cell type of interest in each sample, the values were used for statistical analysis.

The intensity of Prussian blue staining of placental iron storage was subjectively graded between 1 and 5, where 1 indicated minimal staining and 5 indicated high-intensity staining. All the locations in the section that had a positive signal were graded. The mean of the overall grade for each sample was used for further statistical analysis.

### 2.11. Statistical analysis

Mann-Whitney analysis was used to investigate the difference between normotensive, early- or late-onset PE pregnancies. A p-value <0.05 was considered statistically significant. The data is presented as individual values as well as the median. GraphPad Prism 9 (GraphPad Software Inc.) was used for data analysis and graph presentation.

## 3. Results

### 3.1. Description of patient demographics

Clinical demographics for patients included in this study are listed in Tables 1–3 and Supplementary Tables S1–S3, according to the respective analyses. Among all the women, the pregnancies with early-onset PE had significantly lower gestational age at the time of delivery compared to normotensive controls and late-onset PE. This was accompanied by significantly lower fetal birthweight compared to normotensive and late-onset PE, with 33–50% of the fetuses defined as SGA. In addition, maternal BMI was only significantly higher in late-onset and early-onset PE compared to the normotensive group in Table 2. Doppler analyses were available for 44% (Table S2) or 100% (Tables S1 and S3) for early-onset PE patients, and for 50–67% of the late-onset PE patients (Tables S1–S3). It demonstrated 100% presence of bilateral notch, significantly higher pulsatility index (PI) and a significantly higher uterine artery score (UAS) for early-onset PE compared to late-onset PE (Tables S1–S3). The last Doppler analysis before delivery was performed at GW 31 in early-onset PE compared to GW 35–39 for late-onset PE.

### 3.2. Maternal serum ferritin levels are higher early in pregnancy in late-onset PE

To analyze maternal iron homeostasis and bioavailability, maternal serum ferritin and hemoglobin levels were acquired from patient clinical records, measured in clinical blood samples taken during antenatal visits in early pregnancy during late 1st/early 2nd trimester or late 2nd trimester.

**Fig. 1.**
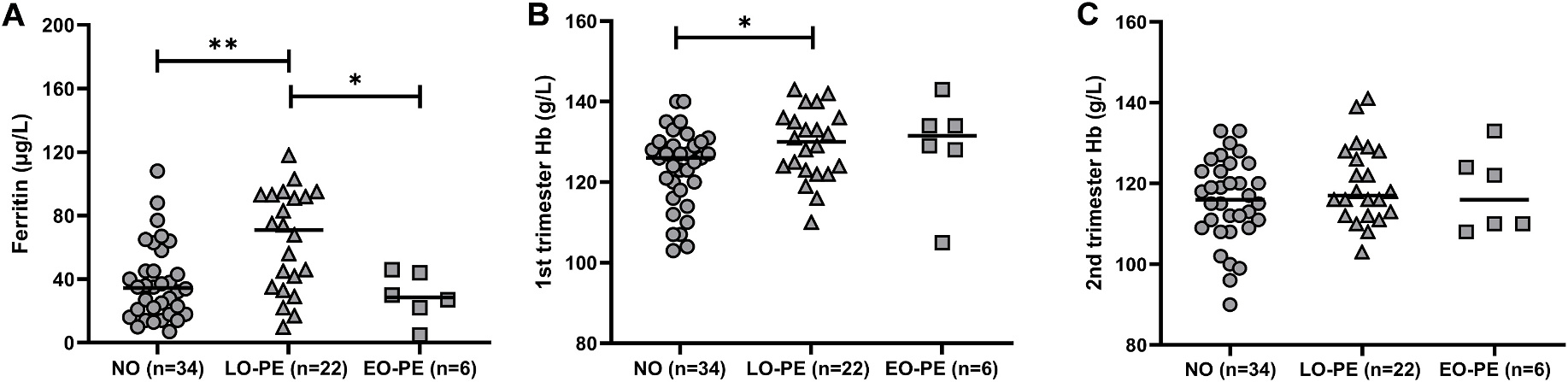
Maternal serum ferritin and hemoglobin levels in normotensive, late-onset, and early-onset PE pregnancies obtained from patient records. (A) Maternal 1st trimester serum ferritin levels. (B) Maternal late 1st/early 2nd trimester serum Hb levels. (C) Maternal late 2nd trimester serum Hb levels. Data is presented as individual values with median for each group. Mann-Whitney test: **p < 0.01, *p < 0.05.

First trimester serum ferritin levels were significantly higher in late-onset PE when compared to normotensive controls (p = 0.002) and early-onset PE (p = 0.019) (Fig. 1A). There was no significant difference comparing early-onset PE and normotensive controls. Maternal hemoglobin levels in first trimester were significantly higher in late-onset PE (p = 0.046) (Fig. 1B), while in late 2nd trimester there was no significant difference between groups (Fig. 1C).

Additionally, information regarding iron supplementation during pregnancy showed that most participants, in all groups, used iron supplements (Table 1). Therefore, no specific correlation was found between maternal iron status, iron supplementation and pregnancy outcome.

### 3.3. Higher ERFE and lower hepcidin levels in maternal plasma in early-onset PE at time of delivery

**Fig. 2.**
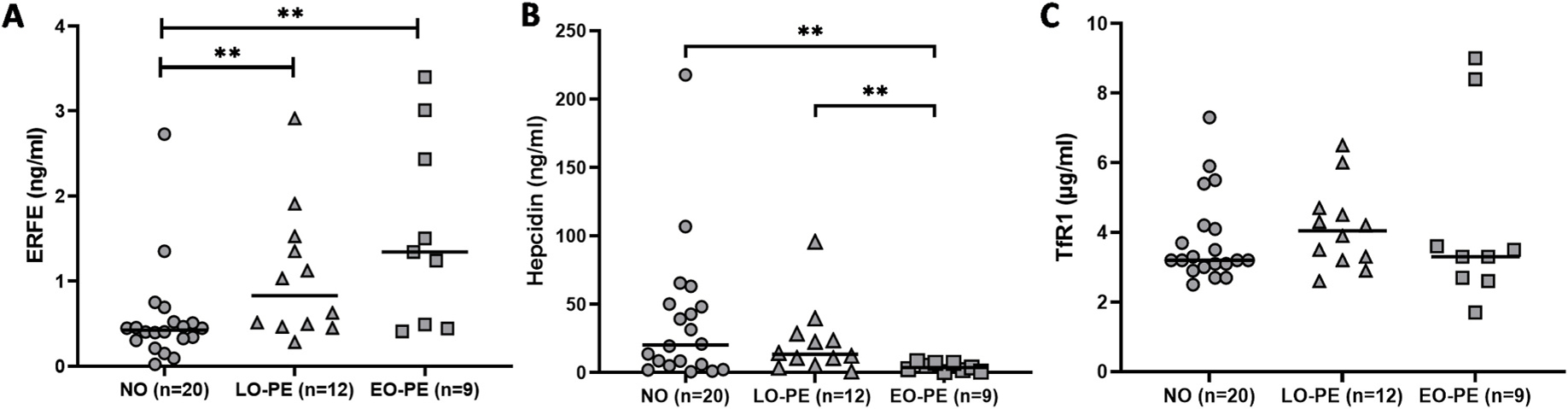
Maternal plasma ERFE, hepcidin and TfR1 levels in normotensive, late-onset, and early-onset PE pregnancies. (A) Maternal plasma ERFE levels at time of delivery. (B) Maternal plasma hepcidin levels at time of delivery. (C) Maternal plasma TfR1 levels at time of delivery. Data is presented as individual values with median for each group. Mann-Whitney test: **p < 0.01.

Levels of ERFE, hepcidin and ferritin in maternal plasma were measured in samples collected at delivery to evaluate maternal hormonal regulation of iron bioavailability. Maternal plasma ERFE levels were significantly higher among both late-onset PE (p = 0.008) and early-onset PE (p = 0.005) compared to normotensive pregnancies (Fig. 2A). Maternal plasma hepcidin levels were significantly lower in early-onset PE compared to both normotensive (p = 0.006) and late-onset PE (p = 0.004) (Fig. 2B). There were no significant differences for maternal plasma ferritin levels between normotensive, early- or late-onset PE pregnancies at time of delivery (Fig. S2A). Analysis of maternal plasma TfR1 levels at this timepoint were used as a marker of maternal iron bioavailability. There were no significant differences in maternal TfR1 between normotensive, early- or late-onset PE pregnancies (Fig. 2C). In addition, maternal plasma levels of the inflammation marker IL-6 showed no significant differences at time of delivery between normotensive, early- or late-onset PE samples (Fig. S2B).

### 3.4. Lower placental TfR1 expression in PE

**Fig. 3.**
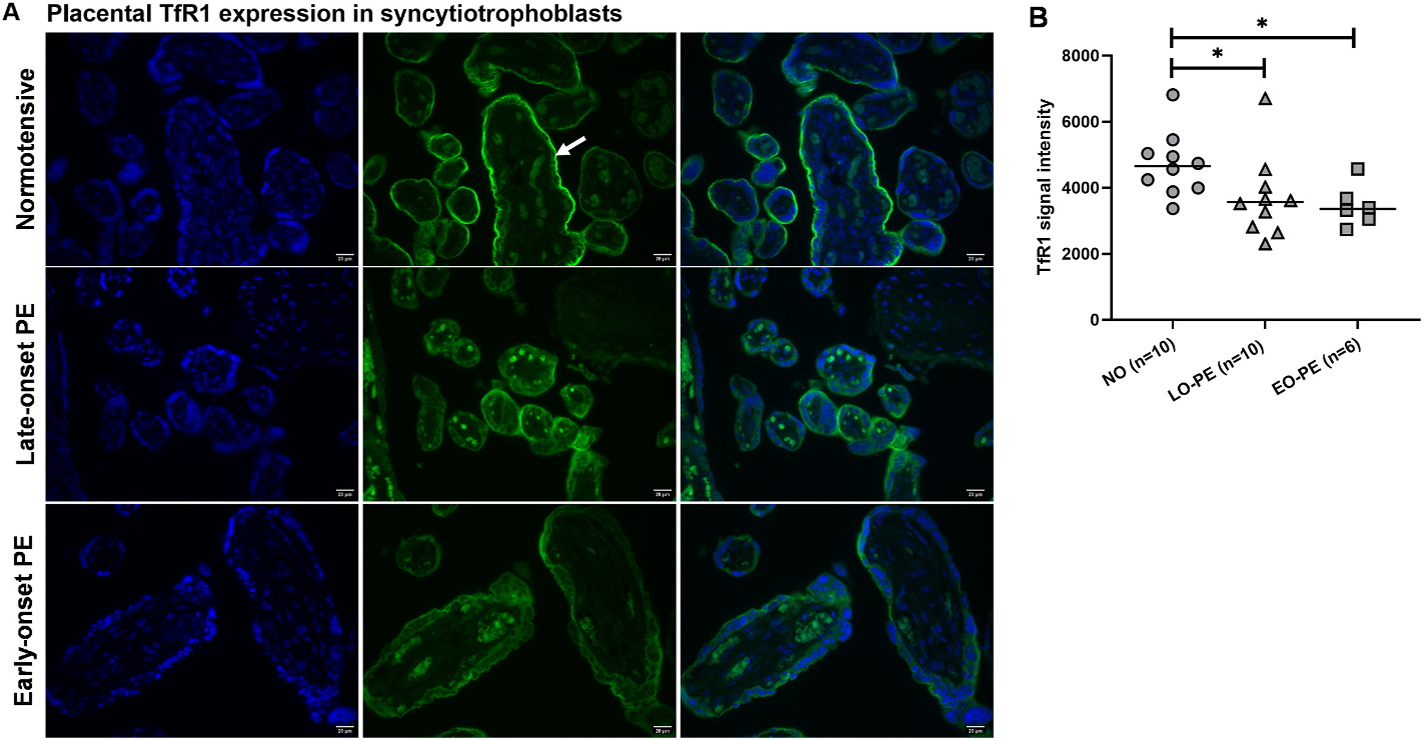
Immunostaining analysis of placental TfR1 protein expression in syncytiotrophoblasts in normotensive, late-onset, and early-onset PE pregnancies. (A) Placental TfR1 expression (white arrow) was localized in the outer membrane. Blue is nuclear DAPI staining, and green is immunofluorescence TfR1 staining. Scale bar: 20 μm. (B) Quantified placental TfR1 signal intensity. Data is presented as individual values with median for each group. Mann-Whitney test: *p < 0.05.

Immunostaining analysis of TfR1 indicated its presence and localization on the outer membrane of the syncytiotrophoblasts in direct contact with the maternal blood (Fig. 3A). Placental TfR1 expression was significantly lower among late- and early-onset PE when compared to normotensive pregnancies (p = 0.018 and p = 0.011, respectively) (Fig. 3B). Negative control images are presented in Supplementary Fig. S1A.

### 3.5. Higher placental FPN expression in early-onset PE

**Fig. 4.**
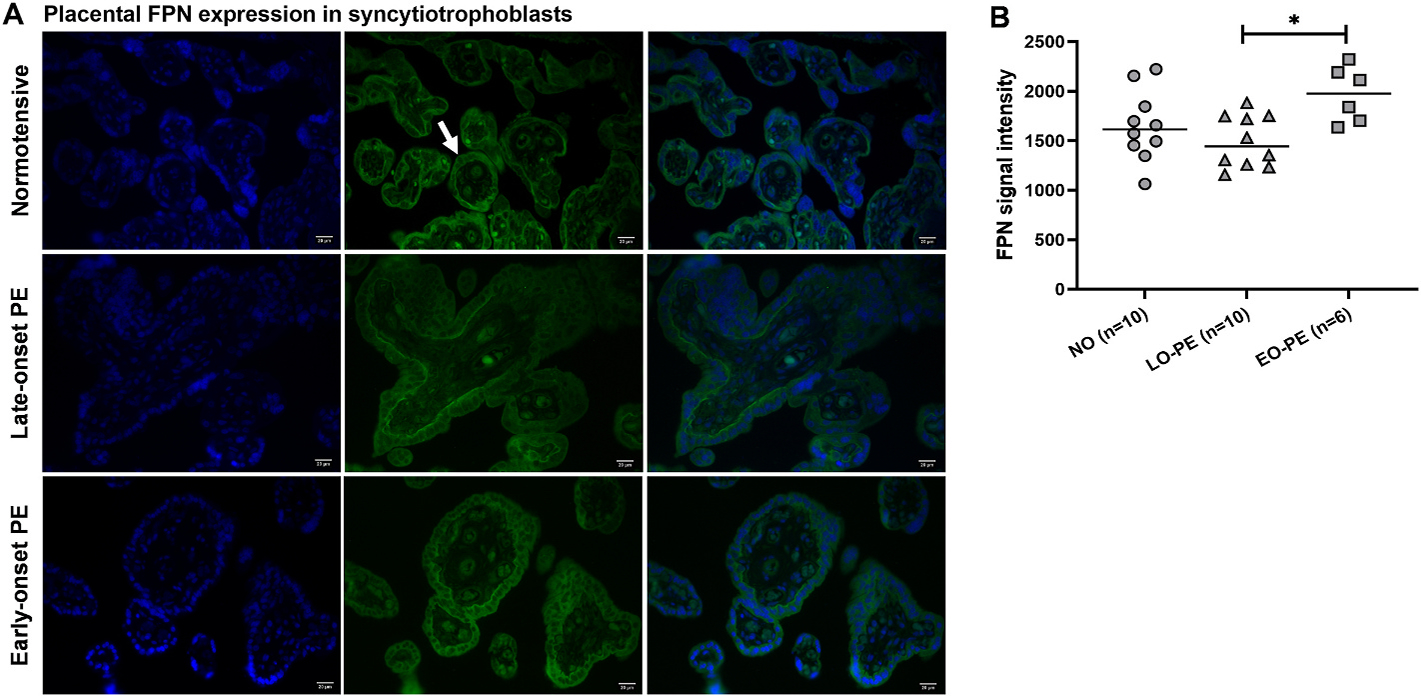
Immunostaining analysis of placental FPN protein expression in syncytiotrophoblasts in normotensive, late-onset, and early-onset PE pregnancies. (A) Placental FPN expression (white arrow). Blue is nuclear DAPI staining, and green is immunofluorescence FPN staining. Scale bar: 20 μm. (B) Quantified placental FPN signal intensity in normotensive and PE pregnancies. Data is presented as individual values with median for each group. Mann-Whitney test: *p < 0.05.

Immunostaining analysis of placental FPN indicated that it was expressed in syncytiotrophoblasts (Fig. 4A). The expression was not different in PE subgroups compared to normotensive controls but was significantly higher in early-onset PE in comparison to late-onset PE (p = 0.022) (Fig. 4B). Negative control images are presented in Supplementary Fig. S1B.

### 3.6. Placental ERFE expression decreases in early-onset PE

**Fig. 5.**
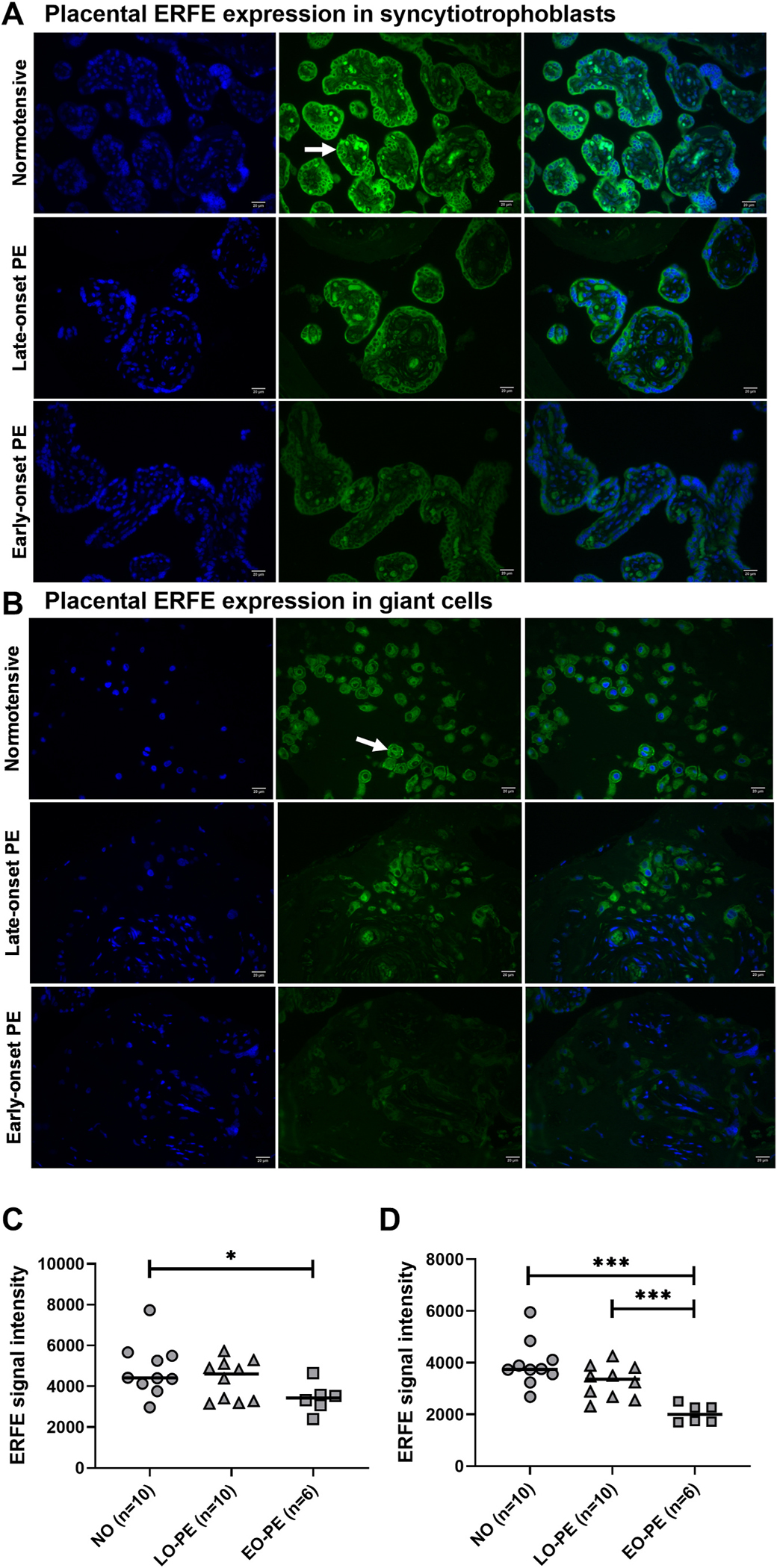
Immunostaining analysis of placental ERFE protein expression in normotensive, late-onset, and early-onset PE pregnancies. (A) Placental ERFE expression in syncytiotrophoblasts (white arrow). (B) Placental ERFE expression in placenta bed giant cells (white arrow). Blue is nuclear DAPI staining, and green is immunofluorescence ERFE staining. Scale bar: 20 μm. (C) Quantified placental ERFE signal intensity in syncytiotrophoblasts. Data is presented as individual values with median for each group. Mann-Whitney test: *p < 0.05. (D) Quantified placental ERFE signal intensity in giant cells. Data is presented as individual values with median for each group. Mann-Whitney test: ***p < 0.001.

Immunostaining analysis of ERFE in the placenta indicated that it was expressed in trophoblasts, in both syncytiotrophoblasts (Fig. 5A, C) and in placenta bed giant cells (Fig. 5B, D). The expression of ERFE in syncytiotrophoblasts was significantly lower in early-onset PE compared to normotensive (p = 0.0002) (Fig. 5C). In placenta bed giant cells, ERFE expression was lower in early-onset PE vs normotensive and late-onset PE (p = 0.031 and p = 0.0005, respectively) (Fig. 5D). Negative control images are presented in Supplementary Figs. S1C and D.

### 3.7. Higher placental iron storage in early-onset PE

**Fig. 6.**
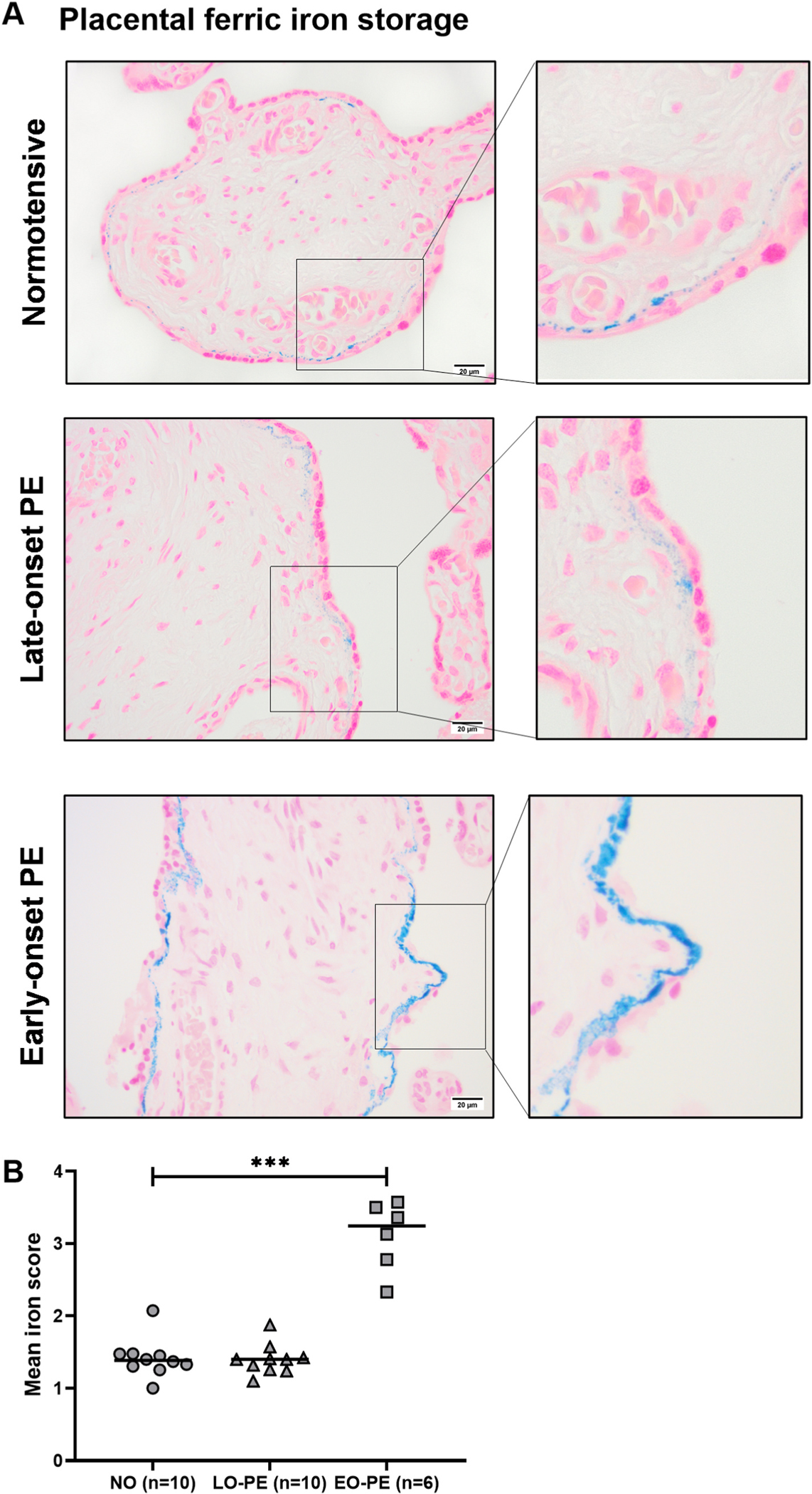
Prussian Blue iron staining in placenta. (A) Detection of placental ferric iron in normotensive, late-onset, and early-onset PE placentas (black arrow). Scale bar: 20 μm. (B) Mean iron scoring for normotensive and PE pregnancies. Data is presented as individual values with median for each group. Mann-Whitney test: ***p < 0.001.

Ferric iron storage in the placenta was analyzed using Prussian Blue staining. Most of the stored iron, particularly in early-onset PE placentas, was detected in the fetal basal lamina under syncytial or cytotrophoblast membranes in the placental villi (Fig. 6A). All areas showing iron staining were scored in each sample (number of areas (mean): normotensive-73, late-onset PE–56, and early-onset PE-27). Mean score for iron storage was 3.2 in early-onset PE which was significantly higher (p = 0.0002) compared to 1.4 for placentas from normotensive and late-onset PE pregnancies (Fig. 6B).

## 4. Discussion

In this study, we investigated the regulation of maternal and placental iron availability and storage, in normotensive and pregnancies complicated by early- or late-onset PE. Focusing on ERFE as a new marker and hormonal regulator of iron, our results indicated altered iron regulation, bioavailability, and placental transport in PE pregnancies.

Establishment of utero-placental circulation triggers pregnancy hypervolemia and affects maternal erythropoiesis in late 1st/early 2nd trimester [26,27]. At this time in pregnancy, our results indicated higher maternal serum ferritin levels in women who developed late-onset PE. These changes may point to an early-onset iron overload or inflammation seen in this group [28] that cannot be detected by late second trimester. Absence of a significant difference in maternal ferritin levels in early-onset PE pregnancies compared to normotensive controls can be, despite the small population size, due to reduced placental perfusion and different cellular mechanisms underlying early-vs late-onset PE.

Erythroferrone is a hormone that is reportedly produced in erythroblasts and is particularly important due to its role in counteracting hepcidin [16]. Hepcidin limits iron availability by suppressing dietary iron uptake and blocking iron mobilization from internal storage [15]. Hepcidin has been the focus of many studies on maternal iron regulation in pregnancy and iron transfer to the fetus [18,19]. However, recent studies suggest a similarly strong role for ERFE [29,30]. Considering hepcidin expression increases as a response to inflammation [31–33], it is difficult to separate this from changes related to iron regulation, especially in pregnancies associated with inflammation such as PE [34–36]. On the other hand, the main known regulator of ERFE is erythropoietin (EPO) [16]. Maternal EPO levels are shown not to be significantly different between normotensive and PE pregnancies [37], making ERFE a very interesting factor to study as a regulator and indictor of maternal iron bioavailability in PE. At time of delivery, maternal plasma TfR1 and ferritin levels were similar in all groups suggesting comparable maternal iron stores. In addition, based on the IL-6 plasma levels, there were no signs of increased inflammation when comparing the study groups. However, higher maternal plasma ERFE levels in both late- and early-onset PE, and lower hepcidin levels observed in early-onset PE suggested a hormonal drive to increase maternal iron bioavailability in these pregnancies, a phenomenon that may underlie the iron overload previously described in PE pregnancies [28]. More studies on a larger sample population could further elucidate these mechanisms in relation to the subtypes of PE and if there is any sexual dimorphism in iron regulation.

Maternal iron bioavailability affects placental iron uptake and transport. Maternal Tf is reported to decrease in PE [36,38] which, along with other factors discussed here, could affect iron bioavailability. Binding of iron loaded Tf to its receptor, TfR1, is the basic mechanism of placenta iron uptake [21,39]. In the placenta, FPN is the main iron transporter that regulates transfer of iron from the syncytiotrophoblasts to fetal vascular endothelial cell basal lamina where it can be stored and further transported into the fetus [21,39]. Altered FPN expression can affect tissue and cellular content of iron both systemically and on a local level [40]. Our results were in line with previous studies showing that placental TfR1 and FPN were mainly expressed on the apical and basal surface of the syncytiotrophoblasts, respectively [20]. The TfR1 expression was lower in placentas from early- and late-onset PE compared to normotensive pregnancies. But FPN expression was higher in placentas from early-onset vs late-onset PE. Cell surface expression of TfR1 is directly affected by iron availability and its expression decreases during iron overload to limit iron uptake [40]. In a mouse model, maternal iron overload led to a decrease in placental TfR1 expression at mid to late gestation (E15.5) [19]. Placental FPN was not affected by maternal iron overload but was downregulated in severe maternal iron deficiency to preserve placental iron [19]. Taken together, the lower placental TfR1 expression in early- and late-onset PE pregnancies may be due to higher maternal iron bioavailability regulated by ERFE and hepcidin. But the increased placental FPN may be regulated by other factors such as the hypoxia-induced signaling pathways [41], known to be activated in PE pregnancies as a result of impaired placental perfusion [42,43]. This is supported by our data showing higher placental FPN in early-onset PE pregnancies that presented with smaller fetuses and reduced placental perfusion according to Doppler analysis, compared to late-onset PE and normotensive pregnancies (Fig. 4 and Table 3).

Following the differences in placenta TfR1 and FPN levels, we explored placental expression of ERFE [16], and placental iron deposits. Our results demonstrated that ERFE was expressed in trophoblasts, in both syncytiotrophoblasts as well as placental bed giant cells. This is the first report of ERFE expression in trophoblasts. Although understanding the specific role of ERFE in trophoblasts requires further investigation, the expression of ERFE in both giant cells and syncytiotrophoblasts were significantly lower in early-onset PE compared to both normotensive and late-onset PE. Further investigation is required to describe the exact role of ERFE in the placenta, but its significance in regulating iron mobilization in both the mother and embryo is prominent [30]. Coincidentally, the significant decrease in placental ERFE was associated with higher placental iron deposit in the early-onset PE compared to late-onset PE and normotensive pregnancies. This can suggest that ERFE plays an important role in regulating placental iron storage, mobilization, and transport, which may also be affected by altered placental perfusion, hypoxia, and FPN expression in early-onset PE. However, it should be considered that the placentas from this group were at an earlier gestational week compared to the other groups. Placental iron deposits decrease by fetal development progression [9], suggesting that gestational age might play a role in the level of placental iron storage and the relevant regulatory proteins. Considering the positive effect of ERFE on iron uptake, mobilization, and bioavailability [16], it would be intriguing to examine whether changes in placental ERFE expression during fetal development may regulate placental iron transport in normal as well as in complicated pregnancies.

The limitation of the study was the low number of early-onset PE cases available. These cases are rarer and account for only 20% of all PE cases. It would have been valuable to confirm the placental ERFE expression by e.g., Western blot or by gene expression. However, placental biopsies are contaminated with both maternal and fetal blood. In addition, PE pregnancies have increased levels of immature red blood cells [44] that express ERFE, which would interfere with total protein or RNA analysis.

In summary, our results demonstrated altered maternal plasma ERFE and hepcidin levels, supporting hormonal deregulation of maternal iron bioavailability in early- and late-onset PE vs normotensive pregnancies that was independent of inflammation. These changes were associated with lower placental TfR1 in syncytiotrophoblasts in both early- and late-onset PE. Additionally, we are the first to report the expression of ERFE in trophoblasts and indicate its decreased levels in early-onset PE placentas. As a regulator of iron mobilization, placental ERFE may play a significant role in controlling iron transport to the fetus, indicating the importance of further investigations to determine its exact role in regulating maternal-placental-fetal iron transport axis.

## Supplementary Material

Suppl. Fig.1

Suppl. Fig.3

Suppl. Fig.2.

## Figures and Tables

**Table 1:** Patient demographics for pregnancies included in maternal ferritin and hemoglobin analyses (Fig. 1). Data is presented as median values (min-max) for each group.

	Normotensive (n = 34)	Late-onset PE (n = 22)^a^	Early-onset PE (n = 6)

Maternal age	29 (19–41)	29 (23–39)	37 (21–40)
Maternal BMI	23 (19–42)	25 (19–35)	25 (20–33)
Gestational age (weeks + days)	39 + 4 (36+3–41 + 5)	39 + 0 (35+4–40 + 4) *	32 + 2 (30+1–34 + 5) ****
Fetal weight (g)	3442 (2345–4959)	3358 (2290–4770)	1531 (1085–2520) ****
SGA (%)	0	5	50
Fetal sex (n)	female (17), male (17)	female (9), male (13)	female (3), male (3)
Iron supplement (%)	76	73	50
Delivery mode	V-34, C-0	V-16, C-6	V-0, C-6

a 2 twin pregnancies. PE = preeclampsia; BMI = body mass index; SGA = small for gestational age defined as weight deviation of 2x Standard Deviations according to Niklasson et al. [23]; V = vaginal delivery, C = Cesarean section delivery.

Mann-Whitney test:

* p < 0.05

**** p < 0.0001.

**Table 2:** Patient demographics for pregnancies included in maternal ERFE and TfR1 ELISA analyses (Fig. 2). Data is presented as median values (min-max) for each group.

	Normotensive (n = 20)^a^	Late-onset PE (n = 12)^b^	Early-onset PE (n = 9)

Maternal age	29 (19–36)	31 (24–37)	33 (21–40)
Maternal BMI	23 (20–31)	25 (19–38) *	26 (20–39) *
Gestational age (weeks + days)	39 + 4 (36+4–41 + 5)	39 + 2 (36+0–41 + 1)	30 + 0 (24+4–34 + 5) ****
Fetal weight (g)	3260 (2566–4845)	3132 (2445–4770)	1420 (450–2520) ****
SGA (%)	0	0	33
Fetal sex (n)	female (7), male (13)	female (7), male (5)	female (4), male (5)
Delivery mode	V-17, C-3	V-8, C-4	V-0, C-9

a 1 twin pregnancy.

b 2 twin pregnancies. PE = preeclampsia; BMI = body mass index; SGA = small for gestational age defined as weight deviation of 2x Standard Deviations according to Niklasson et al. [23]; V = vaginal delivery, C = Cesarean section delivery.

Mann-Whitney test:

* p < 0.05

**** p < 0.0001.

**Table 3:** Patient demographics for singleton pregnancies included in histological and immunostaining experiments. Data is presented as median value (min-max) for each group.

	Normotensive (n = 10)	Late-onset PE (n = 10)	Early-onset PE (n = 6)

Maternal age	28.5 (19–41)	33.5 (26–38)	33.5 (21–40)
Maternal BMI	23 (17–27)	24 (20–34)	24.5 (23–33)
Gestational age (weeks + days)	39 + 0 (36+4–40 + 5)	38 + 4 (36+0–40 + 4)	31 + 3 (26+0–34 + 6) ***
Fetal weight (g)	3617 (2905–4420)	3148 (1925–4546)	1492 (650–2520) ***
SGA (%)	0	10	33
Fetal sex (n)	female (5), male (5)	female (5), male (5)	female (3), male (3)
Delivery mode	V-10, C-0	V-8, C-2	V-0, C-6

PE = preeclampsia; BMI = body mass index; SGA = small for gestational age defined as weight deviation of 2x Standard Deviations according to Niklasson et al. [23]; V = vaginal delivery, C = Cesarean section delivery.

Mann-Whitney test:

*** p < 0.001.

## Abbreviations:

EPO: erythropoietin
ERFE: erythroferrone
FPN: ferroportin
GW: gestational week
IL-6: interleukin 6
LDH: lactate dehydrogenase
PE: preeclampsia
RT: room temperature
Tf: transferrin
TfR1: transferrin receptor 1
