# Supplementary figures and images for: Assessing erythroferrone and iron homeostasis in preeclamptic and normotensive pregnancies: A retrospective study
Source: Placenta. Author manuscript; Available in PMC 2024 Nov 8. (PMC11544558; doi:10.1016/j.placenta.2023.01.008)

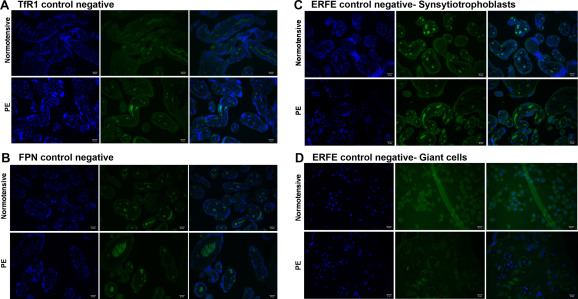

Supplement: Suppl. Fig.1 [file NIHMS2022174-supplement-Suppl__Fig_1.jpg]

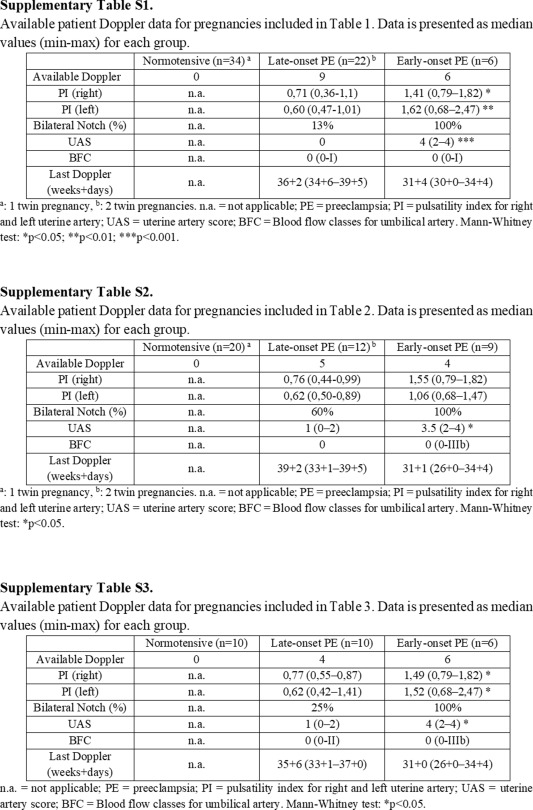

Supplement: Suppl. Fig.3 [file NIHMS2022174-supplement-Suppl__Fig_3.jpg]

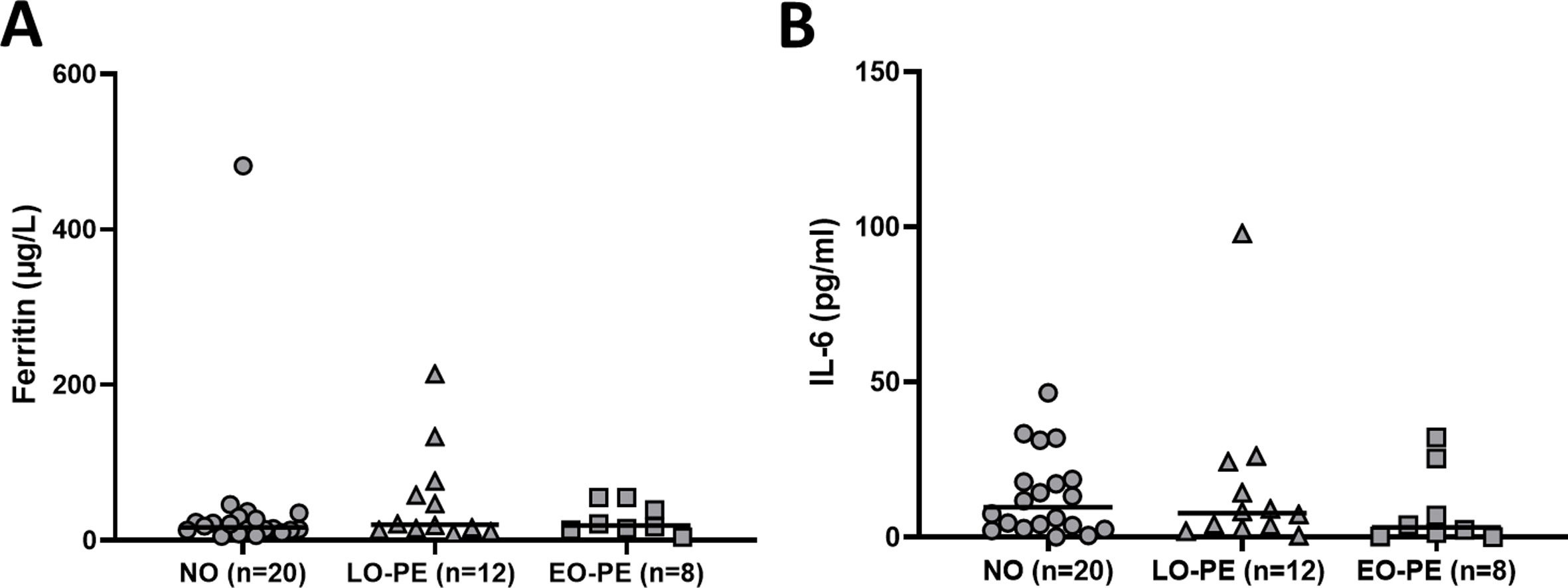

Supplement: Suppl. Fig.2. [file NIHMS2022174-supplement-Suppl__Fig_2_.jpg]
